# Supplementary figures and images for: Inducible lung epithelial resistance requires multisource reactive oxygen species generation to protect against bacterial infections
Source: PLoS One. 2019 Feb 22;14(2):e0208216. doi: 10.1371/journal.pone.0208216 (PMC6386317; doi:10.1371/journal.pone.0208216)

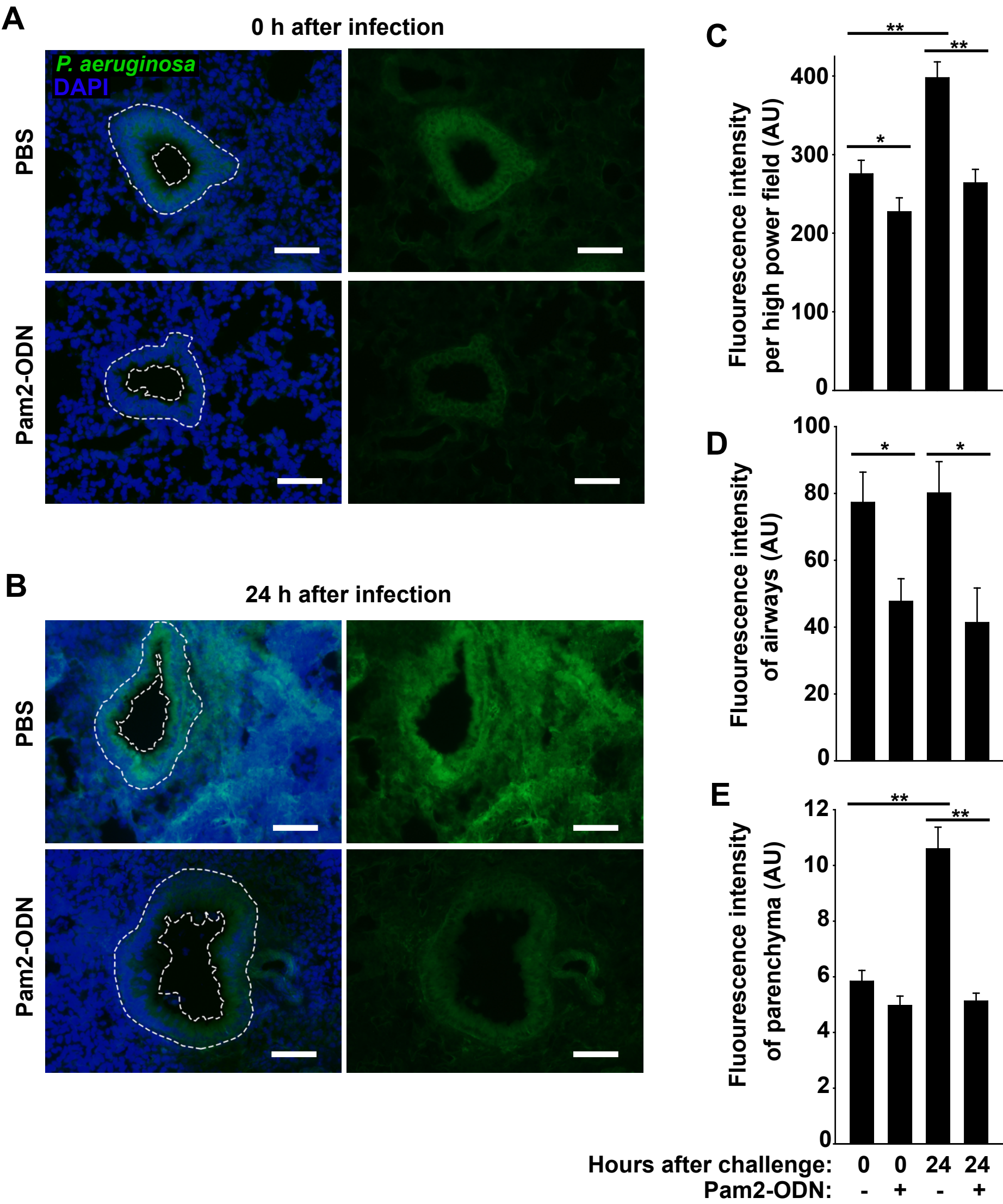

Supplement: S1 Fig — Wild type C57BL/6J mice were treated with Pam2-ODN or PBS (sham) 24 h prior to challenge with GFP-expressing P. aeruginosa. Shown are representative micrographs of 20 μm OCT-embedded lung sections immediately after infection (A) or 24 h after infection (B). Blue, DAPI; green, bacterial GFP; white dashed line, airway delineation; white scale bar, 200 μm. GFP signal in the indicated conditions was then quantified. (C) Mean fluorescence intensity per 20x field. (D) Mean fluorescence intensity of airways contained within the fields examined in C. (E) Mean fluorescence intensity of five stereotyped (174 μm x 174 μm), airway-free boxes from each of the fields examined in C. N = 3 mice per condition, 10 random fields measured per mouse. * p < 0.05, ** p < 0.001. (PDF) [file pone.0208216.s001.pdf]

Ware, Supplemental Figure 2

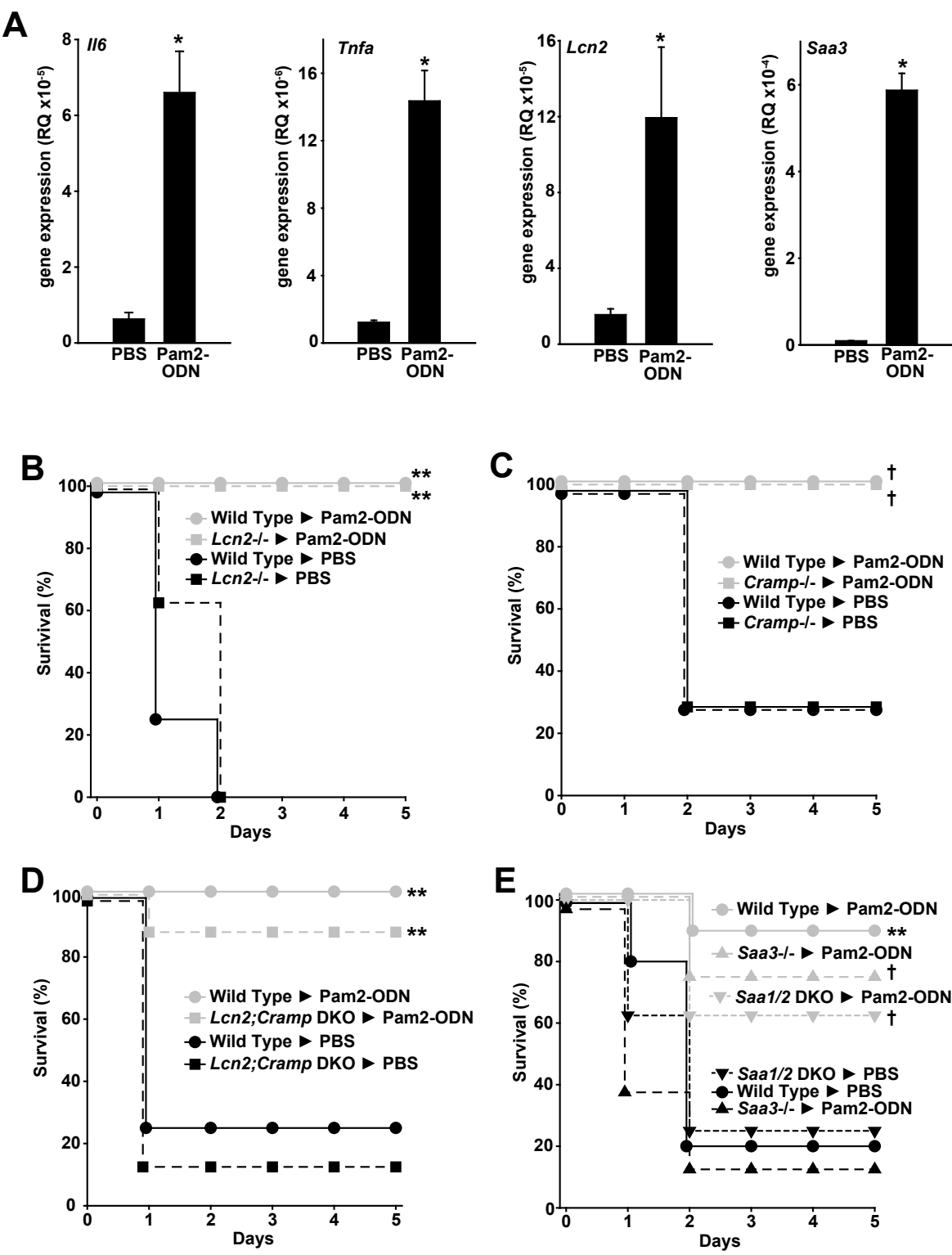

Supplement: S2 Fig — (A) HBEC3kt cells were treated with PBS (sham) or Pam2-ODN for 2 h, then submitted to RT-qPCR for the indicated transcripts. Shown are RQ values for the target transcript relative to 18s gene. Each panel is representative of at least three independent experiments. N = 4–5 samples/condition for all experiments. Wild type or mice deficient in (B) Lcn2, (C) Cramp, (D) Lcn2 and Cramp, or (E) the indicated acute phase SAA genes were treated with PBS (sham) or Pam2-ODN by aerosol 24 h prior to challenge with P. aeruginosa. Shown are survival plots for each challenge. Each panel is representative of at least three independent experiments. N = 8–10 mice/condition. * p < 0.001 vs PBS treated. ** p < 0.007 vs. syngeneic PBS treated. † p < 0.05 vs. syngeneic PBS treated. (PDF) [file pone.0208216.s002.pdf]

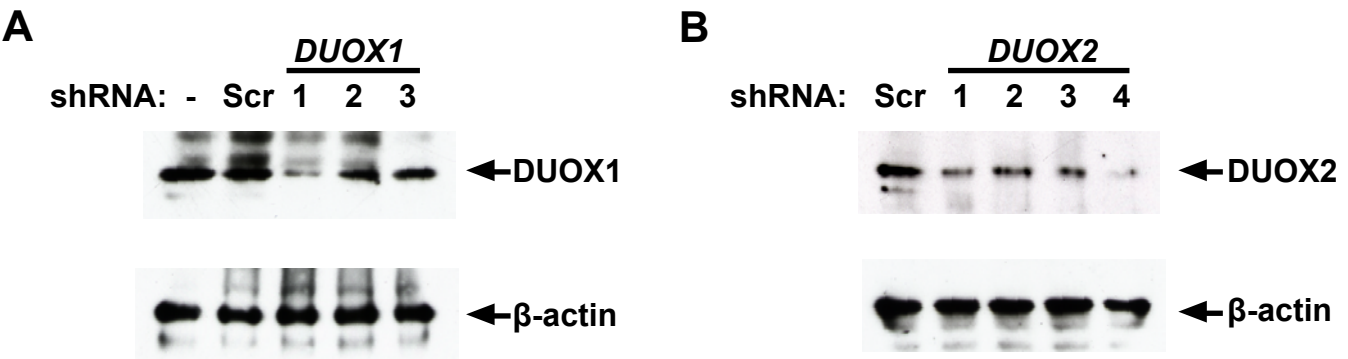

Supplement: S3 Fig — Shown are immunoblots of HBEC3kt cells transfected with scrambled shRNA (Scr) or shRNA targeting DUOX1 (A) or DUOX2 (B), then probed for their respective protein products. Β-actin is shown as a loading control for each sample. (PDF) [file pone.0208216.s003.pdf]
